# Methodologies to Assess the Biodegradability of Bio-Based Polymers—Current Knowledge and Existing Gaps

**DOI:** 10.3390/polym14071359

**Published:** 2022-03-27

**Authors:** João Ricardo Afonso Pires, Victor Gomes Lauriano Souza, Pablo Fuciños, Lorenzo Pastrana, Ana Luísa Fernando

**Affiliations:** 1 MEtRiCS, Departamento de Ciências e Tecnologia da Biomassa, NOVA School of Science and Technology, FCT NOVA, Campus de Caparica, Universidade NOVA de Lisboa, 2829-516 Caparica, Portugal; jr.pires@campus.fct.unl.pt (J.R.A.P.); ala@fct.unl.pt (A.L.F.); 2 International Iberian Nanotechnology Laboratory (INL), Av. Mestre José Veiga s/n, 4715-330 Braga, Portugal; pablo.fucinos@inl.int (P.F.); lorenzo.pastrana@inl.int (L.P.)

**Keywords:** biopolymers, compost, soil, aquatic systems, biodegradation assay, food packaging

## Abstract

Our society lives in a time of transition where traditional petroleum-based polymers/plastics are being replaced by more sustainable alternative materials. To consider these bioproducts as more viable options than the actual ones, it is demanded to ensure that they are fully biodegradable or compostable and that there is no release of hazardous compounds to the environment with their degradation. It is then essential to adapt the legislation to support novel specific guidelines to test the biodegradability of each biopolymer in varied environments, and consequently, establish consistent data to design a coherent labeling system. This review work aims to point out the current standards that can serve as a basis for the characterization of biopolymers’ biodegradation profile in different environments (soil, compost, and aquatic systems) and identify other laboratory methodologies that have been adopted for the same purpose. With the information gathered in this work, it was possible to identify remaining gaps in existing national and international standards to help establish new validation criteria to be introduced in future research and policies related to bioplastics to boost the sustainable progress of this rising industry.

## 1. Introduction

The emergence of different plastics in daily products is directly related to the growth of life’s quality since the 1950s, presenting new opportunities and more social benefits for all mankind [1]. Considering its exceptional intrinsic properties [2], versatility [3], reduced cost [4], and ease of processing [5], it was effortless to achieve the status of most handled material by distinct industries. Among the industries that introduce plastic in their products, the packaging sector stands out globally, with many millions of tons being produced annually, accounting for almost 50% of the total weight. The numbers for the packaging sector are far superior to the other industries, mainly due to single-use plastics, sometimes disposed of after brief periods of working life [6,7]. In spite of all the fascinating aspects undoubtedly linked to conventional plastic, their non-renewable character, unsustainable use, and short lifetime related with their tolerance to degradation cause a serious and realistic environmental problem, bringing out the responsibility to discover innovative and better ways to upgrade the disposal systems [8,9]. Especially in some countries, the lack of viable waste disposal alternatives to the clogged landfills boosts the accumulation of this non-degradable resource in water bodies, leaving a trail of pollution that can be seen from the shoreline to the ocean, causing potential health problems for the population worldwide [1]. In fact, the extensive production of plastics and their non-selective disposal have increased the number of microplastics (MPs) which have been detected in different environments, to the point of being considered already ubiquitous in nature. MPs assimilate the surrounding pollutants (chemicals, heavy metals, microorganisms, etc.) through different types of interactions, increasing the probability of becoming loaded with potential toxic agents. Accordingly, the inhalation and ingestion of these microparticles constitute a relevant exposure route to the health of all living animals who ingest this type of nourishment [10,11].

Regardless of some of the European Union (EU) members having banned landfill applications, there are not yet sufficient alternatives to arouse a paradigm transition, wherein incineration is not an ecological option due to the emission of pollutant gases, in which about 2.8 kg of CO_2_ is evolved in the combustion of 1 kg of plastic. Furthermore, recycling still faces difficulties in separating certain plastics from contaminants, remaining a low-yielding process that needs to be properly adjusted [12,13,14]. Of all the plastic used in food packaging, about 9–10% is recycled, and only half of that percentage survives more than one recycling cycle. These remarkably low rates are associated with factors such as packaging composed by distinct layers of plastic material grades, contaminants present in the packaging (e.g., pigments, inks, and metals), unavailability of suitable waste sorting systems, and deficit of informative sorting and recycling labels [15]. Following the recommendations published in the document “Use of Recycled Plastics in Food Packaging (Chemistry Considerations): Guidance for Industry” by the Food and Drug Administration (FDA), the recommended maximum level of a chemical contaminant present in recycled material, if there is a migration to food, should not exceed 1.5 micrograms/person/day (0.5 parts per billion (ppb) dietary concentration) [16].

Composting is equally an alternative option to overcome the environmental impacts associated with plastics, but the resistance to microbial decomposition severely limits this approach [17,18]. For example, common plastics found in food and beverage packaging, such as polyethylene terephthalate (PET) and high-density polyethylene (HDPE), have an expected life span of 450 and 600 years, respectively [19]. However, undoubtedly, novel insights on waste management and recovery have increased among the industrial sector, the civil and scientific community, formalizing the stimulus for the establishment of a sustainable and bio-based society [20]. The problems exposed above, coupled with consumers’ awareness for healthier and more beneficial products, have guided the replacement of traditional plastics for modern eco-friendly materials. Currently, the use of bioplastics in packaging merely represents values around 1–2% of global plastic packaging sales; however, the United Nations (UN) already considered these biomaterials fundamental to achieving the sustainable development goals [6]. Hereupon, it is essential to continue investing in scientific research and development of biodegradable and bio-based alternatives and adopt contemporary greener policies based on sustainable growth to reduce or even extinguish the negative footprint that petrochemical polymers induce in the environment [21,22]. 

**Figure 1:**
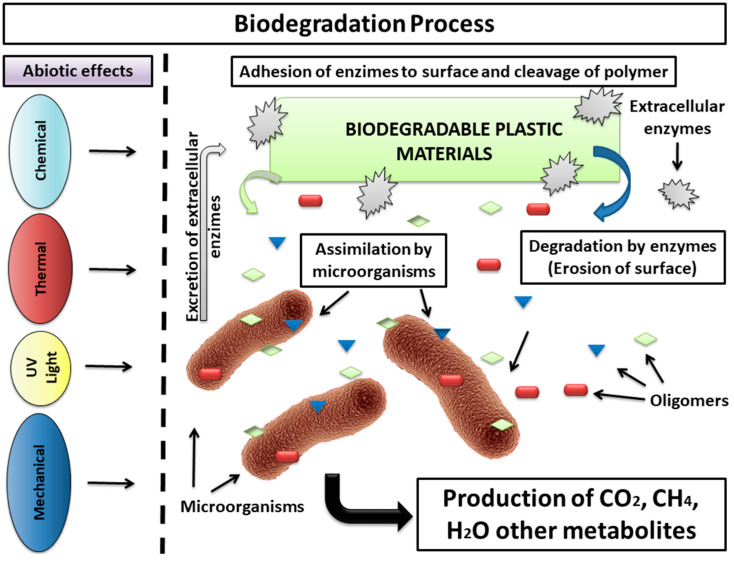
Biodegradation process scheme (Adapted from Souza 2015 [31]).

Bio-based plastics/polymers, recurrently denominated in the literature as bioplastics or biopolymers, have lately been assigned as the logical candidates to substitute conventional plastics due to their renewability, high abundance, accessibility, low cost, reduced toxicity, and biodegradable character [23]. They consist of a chain-like molecule of covalently bonded monomers, in which the difference for polymers resides solely in the prefix “bio”, indicating their biological or renewable origin [12]. Biopolymers can be divided into two vast groups, natural and synthetic, based on their origin. The natural ones are extracted directly from renewable sources, such as proteins (e.g., collagen or gelatin), polysaccharides (e.g., starch, cellulose, or chitin/chitosan), or lipids (e.g., waxes and free fatty acids) or could be produced by microbiological processes such as polyhydroxyalkanoate (PHA) or poly(3-hydroxybutyrate) (PHB). Poly(lactic acid) (PLA) and poly(vinyl alcohol) (PVA) represent some examples of synthetic-produced biopolymers [12,24,25]. Biodegradability is, along with the renewable character, the key point of interest attributed when compared with traditional plastics. It is expected that bio-based plastics can be easily degraded when exposed to sunlight, through photo-oxidation, or fragmentation by the action of living organisms that secrete extracellular enzymes in the presence (aerobic degradation) or absence (anaerobic degradation) of oxygen. The organic chemical substances are then converted into harmless small molecular byproducts, such as carbon dioxide, water, inorganic compounds, methane, and biomass (Figure 1) [23]. Furthermore, some of these elements can also be compostable, at a rate consistent with familiar materials [8]. 

Nevertheless, not all bio-based polymers are biodegradable, and, in opposition, not every fossil-based polymer is non-biodegradable. Indeed, the biodegradation rate differs for each bioplastic, depending on the external environment factors, intrinsic physicochemical properties of the biopolymer, or the characteristics of the filler in the case of blends/composites [2,14,26]. For this reason, it is indispensable to mark an explicit distinction between bio-based plastics and biodegradable plastics. Following the instructions set by the American Society for Testing and Materials (ASTM), bio-based plastic is described as “a plastic containing organic carbon of renewable origin such as agricultural, plant, animal, fungi, microorganisms, marine, or forestry materials living in a natural environment in equilibrium with the atmosphere” [27], while biodegradable plastic is ” a degradable plastic in which the degradation results from the action of naturally-occurring microorganisms such as bacteria, fungi, and algae” [28]. In favor of establishing a generic pattern, the European Bioplastics Association identifies bioplastics as a diverse family of materials, which can be bio-based, biodegradable, or both [29]. It is equally worthy to remark that biopolymer degradation can be hostage from other agents. It was identified that during biological degradation, enzymes produced by micro-organisms are responsible for the degradation. In contrast, in chemical degradation, the agents are oxygen, water, alkaline substances, acids, and solvents. A unique variation of chemical degradation is hydrolytic degradation, and other types of degradation are physical and atmospheric [30].

Thereby, to successfully perform the task of attenuating ecological impact and cut the waste connected with the end of life of single-use plastics, each novel bio-based plastic must be intensively deliberated and optimized earlier than being marketed, ensuring that it is fully biodegradable and that any detrimental ecotoxicological effects emerge from such degradation [21]. This is particularly important when new and innovative solutions with bioplastics are being tested to turn them more commercially available to reduce the dependence of some industries on fossil-based-plastic, such as the food packaging industry. Additionally, to make it feasible for a biopolymer to match the functionality of traditional plastics in food packaging, and as a result go from conception to market, it is pivotal to reinforce their matrix, modifying and upgrading their characteristics. Therefore, another undergoing solution is the incorporation of organic or inorganic nanoparticles to reinforce the film, and the addition of active elements, such as extracts and essential oils, to increase the antimicrobial and antioxidant properties, nonetheless, these new added elements could slower the biopolymer degradation [9]. 

As follows, the aim of this work was to gather the knowledge and discuss: (i) the current standard methodologies that can be applied to test and monitor the biodegradation of the bioplastics; (ii) the effect of environmental conditions, chemical composition, and additives on biodegradability; (iii) the verification of research works that test alternative approaches to analyze the biodegradability of bioplastics in various environments. The information gathered supports the identification of bottlenecks in existing national and international standards used to characterize plastics degradation so that concepts related to bioplastics can be correctly established and categorize the existing limits to bioplastics degradation to identify potential opportunities to boost the sustainable progress of this rising industry.

## 2. Current Biodegradation Assays Methodologies

**Figure 2:**
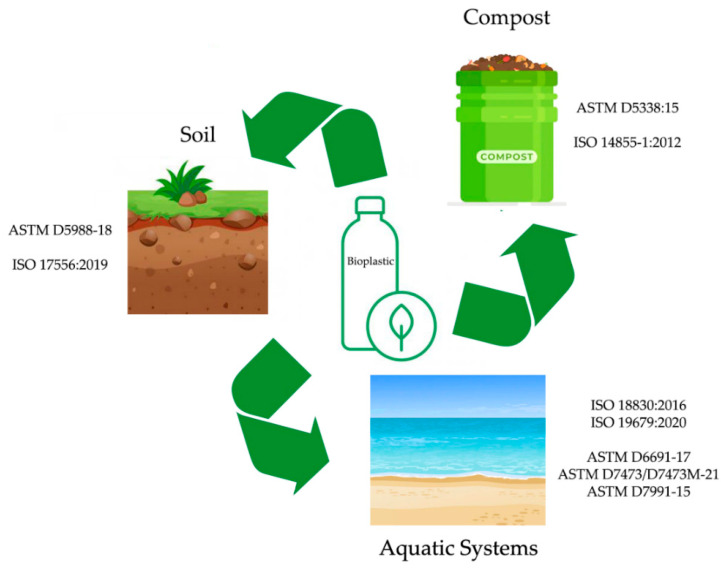
Main standards adopted for assessing the biodegradability of bioplastics.

The methodologies currently used to assess the biodegradability of biopolymers can be organized according to the place where the plastics are being disposed, namely: (i) in soils; (ii) in compost; (iii) in aquatic systems (Figure 2). 

### 2.1. Biodegradation Assays in Soil

Over time, several biological, geological, and hydrologic phenomena shaped the earth’s crust creating new porous land surfaces that usually are defined as soil. The uncertainty generated by the complex number of natural variables means that on our planet it is fairly easy to find soils with uncommon temperatures, pH, moisture, and amounts of oxygen that consequently affect local microbiota activity and composition [32,33]. Under these circumstances, it is tough to find an ecosystem as rich in biodiversity as soils. In just one gram of it, a population of about 10 billion microorganisms that live directly in communion with the existing fauna, containing thousands of bacteria and fungi unique species is established [33]. Biodegradation, as the name implies, is directly related to the degradation of macromolecular chains through the action of microorganisms. Accordingly, the correlation between this process and the vastness of microorganisms in soils promotes a more susceptible and desirable bioplastic degradability study than in other locations [13,34]. 

Examining the literature, it is noted that miscellaneous techniques to evaluate the biodegradation of biopolymers in the soil are being adopted, since currently there are no European or International specifications or criteria settled for this purpose. This is a little bittersweet since some European markets already sell packaging that claims to biodegrade in soil [32]. It is not imperative to follow standards to interpret that a product based on an organic material shows potential to be biodegradable; however, the problem resides in the determination of the exact mass loss and time required by each bioplastic to decompose before receiving that classification [13,34]. For instance, it is perceived that the complete degradation of poly(lactic acid) in natural conditions may take up to several years [35] and it takes some time to start degrading, as demonstrated in an eleven-month test performed in the Mediterranean soil environment by Rudnik and Briassoulis [36]. Yet, a bio-based film made from starch and poly(vinyl alcohol) achieved 90% biodegradation within 28 days using a similar soil burial method [37]. In view of this, it has to be taken into account that each biopolymer is biodegraded in a specific way, reckoning variable intrinsic factors such as crystallinity, chemical structure, molecular weight, surface area, and crosslinks, and likewise is limited by some environmental soil factors such as temperature, moisture, pH, and microbial composition [13,18]. Since the bioplastic definition is so broad, it is required to define proper legislation that indicates whether the “biodegradable” categorization is ordinary to all or differs individually. 

It is imperative to interrogate how the biodegradability of these novel materials can be measured and labeled [38]. Essentially, while any specific guidelines have been defined, the scientific community is majorly following two soil conditions for the assembly of biodegradation processes. If the aim is only to determine the intrinsic biodegradability instead of studying the secondary toxicity effects of it in soil, the laboratory assays seem to be the conventional choice due to a question of time. Indeed, following ISO or ASTM standards previously defined to test the biodegradation of traditional plastics, the samples are prepared under controlled parameters, which optimizes the process [38,39,40,41]. On the other hand, if it is intended to perform a realistic simulation, the biopolymer should be directly buried in outdoor soil respecting the local environmental conditions [18,42]. However, it is equally meaningful to bear in mind that these two methodologies must be complementary. Despite ensuring repeatability, standardized tests are designed to trial under optimal conditions of biodegradation, and these contrast with the sub-optimal conditions (scarce nutrients, insufficient water activity, low temperature, and reduced gas exchange) found in real soils that would constrain the adequate growth of microbes, hence slowing down the biodegradation response [32,38]. 

The most actual standard methodologies for biodegradation of plastics in soil are ASTM D5988-18 “standard test method for determining aerobic biodegradation of plastic materials in soil” [43] and ISO 17556:2019 “plastics—determination of the ultimate aerobic biodegradability of plastic materials in soil by monitoring the oxygen demand in a respirometer or the amount of carbon dioxide evolved” [44]. Briefly, ASTM D5988-18 measures the carbon dioxide developed by microorganisms as a function of time of exposure, thus measuring the degree of biodegradability relative to a reference material. Similarly, the principle behind the ISO 17556:2019 yields the optimum rate of biodegradation of plastic material in a test soil by controlling the oxygen consumption or the carbon dioxide production [41]. For both cases, the two standards research settings are reasonably simple and similar as stated in Table 1. Basically, the bioplastic sample is buried in closed vessels containing previously prepared soil. Subsequently, the vessels are subjected to a temperature range favorable to the growth of mesophilic microorganisms jointly with optimal conditions of moisture and oxygen. Although both indicate the same temperature range, ISO 17556:2019 establishes an ideal temperature of 25 °C. 

**Table 1:** Some soil technical specifications for each standard.

Specifications	ASTM D5988-18	ISO 17556:2019
Type	The soil should be natural and fertile, preferably “sandy loam” collected from fields and forests not exposed to pollutants. The soil used should be a mixture of natural and fertile soils collected from the surface layers of at least 3 diverse locations.	Natural soil from fields and/or forests may be used as an inoculum to simulate biodegradation in a specific natural environment.
Particle size	Less than 2 mm	Less than 5 mm
Room Temperature	20 − 28 ± 2 °C	20 − 28 ± 2 °C but in preference 25 °C
pH	6–8	6–8
Moisture holding Capacity (MHC)	80–100%	40–60%
Running Time	Should not exceed 6 months	Should not exceed 6 months but may be extended up to 24 months
Reference Material	Cellulose or starch	Microcrystalline cellulose powder, ashless cellulose filters or poly(3-hydroxybutyrate)
Validation Criteria	The test is considered valid if after 6 months more than 70% biodegradation is achieved for the reference material and if the amount of CO_2_ evolved from the control reactors is within 20% of the mean at the plateau phase or at the end of the test.	The test is considered valid if the degree of biodegradation of the reference material is more than 60% at the plateau phase or at the end of the test and if the BOD (biological oxygen demand) values of or the amount of CO_2_ evolved from the controls are within 20% of the mean at the plateau phase or at the end of the test.

**Table 2:** Recent methodologies to monitor biodegradation in soil.

Biopolymer *	Soil Conditions	Methodology	Ref.
Chitosan/Corn cob	Laboratory-controlled conditions	ASTM D5988	[39]
PHBV/Olive Pomace	Laboratory-controlled conditions	ASTM D5988	[40]
Cellulose based	Laboratory-controlled conditions	ASTM D5988	[51]
PBAT/Nanocellulose	Laboratory-controlled conditions	ASTM D5988	[52]
Polyurethane (PU)/Starch	Laboratory-controlled conditions	ASTM D5988, morphology and chemical characterization, visual analysis	[48]
Starch/Nanocellulose	Laboratory-controlled conditions	ISO 17556	[53]
Poly(lactic acid) (PLA)/Glycerol	Laboratory-controlled conditions	ISO 17556	[54]
Mater-Bi (Mixture of PBAT, starch, and additives)	Laboratory-controlled conditions	ISO 17556, ecotoxicological analysis	[55]
PHA, PBS, and PBAT/PLA	Laboratory-controlled conditions	ISO 17556, microrganismo characterization	[41]
PHB mixed with natural fillers	Laboratory-controlled conditions	Mass Loss	[47]
Starch/Nanocellulose	Outdoor soil conditions	Mass Loss	[42]
Poly(vinyl alcohol) (PVA)/Starch	Outdoor soil conditions	Mass Loss	[45]
PVA/Starch	Laboratory-controlled conditions	Mass loss, biofilm area, soil characterization, and visual analysis	[37]
PCL, PHB, PLA, and PBS	Laboratory-controlled conditions and outdoor soil conditions	Mass loss, mechanical properties, and microorganism characterization	[49]
Starch based	Laboratory-controlled conditions	Mass loss, morphology analysis, and mechanical properties	[56]
PBS/Sugarcane Fiber	Laboratory-controlled conditions	Mass loss, morphology analysis, and thermal characterization	[57]
PHA	Outdoor soil conditions	Mass loss and morphology and chemical analysis	[50]
PLA and PLA/Starch	Outdoor soil conditions	Mass loss, morphology and chemical analysis, and thermal characterization	[18]
PVA/Starch and PVA/Starch mixed with different natural fillers	Laboratory-controlled conditions	Mass loss, morphology and chemical analysis, and soil characterization	[58]

* Poly(3-hydroxybutyrate-co-3-hydroxyvalerate) (PHBV), Poly(butylene adipate-co-terephthalate) (PBAT), Polyhydroxyalkanoate (PHA), Poly(1,4-butylene succinate) (PBS), Poly(3-hydroxybutyrate) (PHB), Polycaprolactone (PCL), Poly(1,4 butylene) succinate (PBS), and Poly(vinyl alcohol) (PVA).

These standards should not merely serve as a guide but also as base methods for possible new approaches. Based on this premise, Šerá et al. [41] used ISO 17556:2019 as the basic methodology to test an innovative method that allows the acceleration of biodegradation of slowly degradable polyesters in the soil, raising the control temperature from 25 °C to 37 °C to decrease the analysis time. A moderate increase in temperature aimed to increase the number of extremophilic species but without a drastic change in microorganisms existing at 25 °C, and therefore, be liable for assessing ultimate biodegradation under representative conditions of ambient temperature. In another study, Pischedda et al. [38] observed the effect of temperature in the biodegradation of bioplastics in soil following ASTM D5988-18. They used pellets of a commercial biodegradable plastic material to be tested at 28, 20, and 15 °C. On balance, it was possible to draw a parallel between the temperature and the speed of biodegradation. These two recent studies marked an initial phase towards deploying alternative methodologies to simulate field dissipation kinetics contemplating the effects of soil temperature. 

A parameter that still generates many doubts is the running time, raising some valid questions before setting up the experiment. Both ASTM D5988-18 and ISO 17556:2019 state that the test should not exceed six months, but what should represent the amount of biodegradation that the sample must show to consider the test finished? Should there be complete biodegradation or is it acceptable to merely go beyond the validation values of the materials used as reference? In addition, in which days the bioplastic degradation evolution should be assessed? In two different works, in which was used poly(vinyl alcohol)/starch blended, different experiment designs were tested. It should be registered that the tests were carried out under the laboratory simulated conditions without ensuing any of the standards previously presented, and both evaluated the biodegradation through weight loss percentage over time. One decided that the samples should be collected every 7 days for up to 35 days [37], while the other determined that the samples should be unburied every 10 days for up to 50 days [45]. Sen and Das [37] noted almost total film biodegradation within a period of 35 days, while Kaur et al. [45] registered 96.6% weight loss after 50 days. Adopting these two works as examples, this slightly illustrates the complexity of knowledge that presently can be found in the literature. The biodegradability of similar biomaterials is being tested differently and, therefore, sustaining a broad range of values. This lack of uniformity constitutes a giant challenge of how the authentic biodegradation values for each biomaterial should be established. It has already been mentioned that numerous variables condition affects the polymer biodegradation, but the present standards are limited to encompass all of them. A synergic approach employing various methodologies should be followed when dealing with the biodegradation of bioplastics [46]. Novelty studies are performing extensive assessments and thus presenting more consistent values of biodegradability in soil (Table 2).

Mass loss is the most basic and widely used biodegradation index. Typically, the analysis of this specification is conducted through experimental measurement that consists of collecting samples at different times, drying until achieving a constant weight [42,45,47]. Complementary, another way to quantify the mass loss is through molecular weight measurement using gel permeation chromatography [18]. The morphology analysis with scanning electron microscopy (SEM) is a good complement assay that allows to state the effect of the ingress of water in the biopolymer, which is called surface erosion [48]. Moreover, with this observation, it is possible to analyze and identify the microorganisms that grow superficially during the degradation process. Šerá et al. [41] identified a massive mesh of fungal filaments and fungal spores coating the entire surface of fast degrading materials (PHA and PBS). In opposition, the authors discovered that slowly degradable polymers (PBAT/PLA) and experimental polyester network (denominated ICL-PN) were uncovered with microorganisms. Al Hosni et al. [49] also observed that a range of microorganisms has grown in distinct polymers, taking advantage of them as nutrients, particularly when under starvation and lacking essential nutrition, therefore representing a crucial role in the polymer biodegradation. Subsequently, to improve the characterization, rRNA gene sequencing was performed to identify the fungal isolates. Besides the morphology, the biopolymer surfaces’ chemical composition should also be evaluated. Spectroscopy was used to estimate the biodegradation development through the changes in specific functional groups of the bioplastics [46]. Tai et al. [48] and Sabapathy et al. [50] used Fourier transform infrared spectroscopy (FTIR) to measure the level of degradation, observing the shift and also intensity of specific infrared peaks. 

In another intriguing study, Ibrahim et al. [56] compared the variations in the mechanical properties with the biodegradation behavior of starch-based composites after being reinforced with different lignocellulosic fibers. This is helpful to determine the influence of the filler type in biopolymer’s degradation process. The team discovered that biocomposites’ tensile strength and modulus of elasticity decreased more than 50% during the first week, and then further gradual deterioration inevitably took place until the end of the experiment. 

Before ending this prerogative of ideas, it is worth recalling something mentioned in the introduction. To consider bioplastics as a viable option to one day replace traditional plastics it is necessary to ensure that they are fully biodegradable and that no harmful ecotoxicological effects result from such degradation. Once, during degradation, the biopolymer repeatedly breaks down and drops many different elements to the soil, with the released molecules fulfilling a significant role in plant metabolism, either inhibiting or stimulating flora growth; consequently, it is fundamental to consider ecotoxicological assessments. Seed germination and plant growth bioassay are the most frequent techniques used to complete this evaluation [59]. The *modus operandi* is simple, the biopolymer is buried in soil in an incredibly high dose and suffers biodegradation. From time to time, a sample of the soil is tested with bioassays for ecotoxicity herein with a control sample not exposed to the biopolymer and a control sample exposed to a GRAS (generally recognized as safe) substance, such as cellulose. To rule out negative ecotoxicity effects, no significant difference must be perceived between the test samples and control samples [56]. Still, regarding soil quality, another fascinating topic to be studied in the future is the effect that the degradation of biopolymers can have on the remediation of soils contaminated with heavy metals. It is known that, for example, chitosan demonstrates excellent metal-binding properties [60]. Why not combine the useful with the pleasant and enhance the application of bio packages at the end of life by depositing them in contaminated soil?

Briefly, it is mandatory to establish a European or international standard that helps to complement the already existing ISO and ASTM standards so that it is possible to characterize with more certainty the biodegradability of each biopolymer that is entering the market. Only then will it be possible to state with certainty which are the best techniques for depositing these in soils to guarantee total and safe biodegradability. 

### 2.2. Biodegradation Assays in Compost

Composting is an aerobic method of solid waste management, wherein the presence of microorganisms, under controlled conditions, biodegradable materials are biologically decomposed into humus, which is a good nutrition source for strengthening soil productivity and agricultural yield. This process may be incredibly helpful in the disposal problem and to diminish greenhouse gases emissions since the compost is a beneficial organic amendment and substrate that can be reintroduced into the economic system [17,61,62,63]. 

Analyzing the most recent scientific data is possible to verify that a vast majority of the assays on biopolymers degradation under aerobic composting conditions have been following more standardizations than in soils once these tests are mainly being carried out at laboratory level. As perceived in Table 3, the most adopted standards are the ISO 14855-1:2012 “determination of the ultimate aerobic biodegradability of plastic materials under controlled composting conditions—method by analysis of evolved carbon dioxide—Part 1: General method” [64] and, analogously, the American Society for Testing and Materials has the ASTM D5338-15 “standard test method for determining aerobic biodegradation of plastic materials under controlled composting conditions, incorporating thermophilic temperatures” [65]. Both determine the ultimate aerobic biodegradability (means by which microorganisms entirely consume a chemical or organic substance in the presence of oxygen) of plastics based on organic compounds under controlled composting conditions by measuring the percentage conversion of the carbon into carbon dioxide and the degree of disintegration of the plastic at the end of the test. 

The standards mentioned above were designed with the intention of simulating typical aerobic composting conditions, by exposing the test material to an inoculum derived from the organic fraction of solid mixed municipal waste (Table 4). The composting takes place in an environment wherein temperature, aeration, and humidity are accurately monitored and controlled. The percentage of CO_2_ involved is quantified through acid-base titration or by employing a direct measurement such as infrared or gas chromatography. Additionally, the ISO 14855 has a Part 2, the ISO 14855-2:2018 [66], differing from the first part by implementing a gravimetric method to measure the biopolymer mineralization. The material sample disintegration is estimated comparing the total dry solids of the initial test amount with the retrieved fractions of material superior to 2 mm. 

Equally, as seen formerly for soils, the existent standards are still limited and the conditions disparities between home and industrial composting may lead to a significant difference in the biopolymer degradation character [13]. The fragmentation emerges quicker in an industrial composter because thermophilic temperatures are most easily achieved on a large scale and constitute less risk of microplastics exhibition to the environment as the degradation occurs within a sealed and controlled system. In opposition, it is much more difficult to achieve these temperatures in small-scale residential composting units, often referred to as “backyard” or “home” composting, and the toxicological effects on the environment are by far distinct [3]. Until now, no international standards have been presented concerning specifications for domestic compostability. However, some national grades, mostly based on EN 13432 “requirements for packaging recoverable through composting and biodegradation—test scheme and evaluation criteria for the final acceptance of packaging” [67], have been implemented with the aim of certifying the home compostability of bioplastics. Moreover, TÜV AUSTRIA BELGIUM and DIN CERTCO provide certification for home compostability following the Australian standard AS 5810 “biodegradable plastics—biodegradable plastics suitable for home composting” [68]. To characterize the biopolymer in a less idealized and more realistic composting environment, the ISO 16929:2021 “determination of the degree of disintegration of plastic materials under defined composting conditions in a pilot-scale test” [69] could represent the most adequate alternative. This one determines the degree of disintegration of materials in a pilot-scale test under defined composting conditions, being much more representative of microbial populations than laboratory-scale setups.

**Table 3:** Recent methodologies to monitor biodegradation in compost.

Biopolymer *	Compost Origin	Methodologies to Monitor Biodegradation	Ref.
PLA, PLA/Chitosan, PLA/Nanocellulose, and PLA/Gum	Food waste	ASTM D5338, mass loss, morphology and chemical analysis, thermal characterization, contact angle, and microorganism characterization	[63]
PVA PVA/Nanocellulose	MSW	ASTM D5338, visual analysis, mass loss, morphology and chemical analysis, thermal characterization, and compost quality (including physico-chemical parameters and ecotoxicological analysis)	[17]
PHA-based	MSW	ASTM D5338	[70]
Starch/Carboxymethyl Chitosan	Comercial	ASTM D5338	[71]
Chitosan	Not Specified	ASTM D-5338 and ISO 14855-1, visual analysis, morphology and chemical analysis, thermal characterization, and microorganism characterization	[61]
PVA/Starch	MSW	ISO 14855-1, ISO 20200, visual analysis, morphology analysis, thermal characterization	[72]
Nano-reinforced PLA	MSW	ISO 14855-1, ISO 20200 with adaptations of ISO 16929, visual analysis, and compost quality (including physico-chemical parameters and ecotoxicological analysis)	[73]
Gelatin, Chitosan, and/or Sodium Caseinate based	Mature compost (10%) mixed with synthetic biowaste (contained sawdust (40%), rabbit food (30%), starch (10%), sugar (5%), oil (4%), and urea (1%))	ISO 20200, visual analysis, mass loss, and chemical analysis	[62]
PHA based	Mature compost (10%) mixed with synthetic biowaste (contained sawdust (40%), rabbit food (30%), starch (10%), sugar (5%), oil (4%), and urea (1%))	ISO 20200	[70]
PLA and PLA/Silica	Not speficied compost fermented with biomass (2:1)	Modified ISO 17556	[74]
PLA, PLA/TAC, and PLA/PHB/TAC	MSW	CO_2_ concentration, mass loss, chemical analysis, and thermal characterization	[75]
PCL, PHB, PLA, and PBS	Comercial	Mass loss, mechanical properties, and microorganism characterization	[49]
Starch/Montmorillonite	Not Specified	Mass loss and ecotoxicological analysis	[76]
Nano-reinforced PLA	MSW	Mass loss and microorganism characterization	[77]

* Poly(lactic acid) (PLA), Poly(vinyl alcohol) (PVA), Polyhydroxyalkanoate (PHA), Plasticizer triacetine (TAC), Poly(3-hydroxybutyrate) (PHB), Polycaprolactone (PCL), Poly(1,4 butylene) succinate (PBS), and municipal solid waste (MSW).

**Table 4:** Inoculum technical specifications for ASTM D5338:15 and ISO 14855:2012.

Specifications	ASTM D5338:15	ISO 14855-1:2012
Inoculum	Industrial compost soil that has a maturity level of 2–4 months	Stabilized, mature compost derived (four-month old), if possible, from composting the organic fraction of solid municipal waste
Particle size	-	0.5 cm to 1 cm
Room temperature	Incubated in the dark at 58 °C ± 2 °C	Incubated in the dark at 58 °C ± 2 °C
pH	7.0–8.2	7.0–9.0
Total dry solids content	between 50% and 55% of the wet solids	between 50% and 55% of the wet solids
Volatile solids content	To produce more than 50 mg but less than 150 mg of CO_2_ per gram of volatile solids after 10 days of incubation	No more than about 15% of the wet or 30% of the dry solids
Moisture holding capacity (MHC)	50%	50–60%
Running time	Minimum of 90 days and should not exceed 6 months	Minimum of 90 days and should not exceed 6 months
Reference material	Celullose	TLC (thin-layer chromatography)-grade cellulose with a particle size of less than 20 μm
Validation criteria	If: (a) more than 2 g of volatile fatty acids per kilogram of dry matter in the composting vessel is formed, the test must be regarded as invalid; (b) a minimum of 70% for cellulose within 45 days is not observed with the positive reference, the test must be regarded as invalid and should be repeated, using new inoculum; (c) the deviation of the percentage of biodegradation of the positive reference is greater than or equal to 20% at the end of the test, then the test shall be regarded as invalid.	The test is considered as valid if: (a) the degree of biodegradation of the reference material is more than 70% after 45 days; (b) the difference between the percentage biodegradation of the reference material in the different vessels is less than 20% at the end of the test; (c) the inoculum in the blank has produced more than 50 mg but less than 150 mg of carbon dioxide per gram of volatile solids after 10 days of incubation.

Two other standards were developed for more embracing purposes, the ASTM D6400-21 “standard specification for labeling of plastics designed to be aerobically composted in municipal or industrial facilities” [78] and ISO 17088:2021 “plastics—organic recycling—specifications for compostable plastics” [79]. Besides the evaluation of biodegradability through CO_2_ measurement and disintegration, the ASTM D6400 additionally includes elemental analysis, plant germination (phytotoxicity), and mesh filtration of the resulting particles. In fact, if it is only intended to perform a biodegradation test without the extensive range of compost analysis, ASTM D5338 is enough. In ISO 17088:2021, apart from the intention of study biodegradability and disintegration such as in ISO 14855-1:2012, this normative equally has as purpose the evaluation of negative consequences on the composting process and facility and negative effects on the quality of the resulting compost, including the presence of high levels of regulated metals and other harmful components. Both agree on the identification parameters for material to be considered a compostable material. The polymer should pass in the toxicity tests, and after 84 days less than 10% of the initial weight of the material should disintegrate into fragments that can go across a 2 mm sieve, a further 90% of the organic carbon (absolute or relative) shall be converted to CO_2_ after 180 days. A summary of the presented methodologies can be found in Table 5.

The biopolymer biodegradation assessment is an expansion study field still pursuing uniformity, and as such, the addition of these tests in scientific investigations represents a heroic attempt to prove the outstanding features presented by the prefix “bio” when compared with traditional plastics. Nonetheless, it is equally visible that only a few numbers of studies manage to achieve a complete approach to the subject. Biodegradability tests, in both soil and compost, are complex, extensive, and expensive, and few research teams have laboratory conditions to execute them, most often limiting themselves to following existing standards with the addition of one or another characterization. ASTM D6400 and ISO 17088 are the most comprehensive standards, but they still lack some fundamental characterizations. As follows, it is essential to study the degradation of the biopolymer at various levels and under a multiplicity of environmental conditions to be able to accurately establish complete guidelines. Acknowledging the actual barriers in the following paragraphs, some interesting results of a set of recent articles that assess different biopolymer blends biodegradation from unique perspectives are presented, confirming the importance of complementing the standardization with the study of other parameters.

A biodegradable material is not necessarily compostable. As seen in the compostability requirements stated in ASTM D6400 and ISO 17088, a material to be considered compostable must not only respect certain biodegradability and disintegration rates but also demand to pass in the environmental safety tests. The methods for evaluating the ecotoxicity of compostable polymer materials are typically based on the use of plants, soil fauna (earthworms), aquatic fauna (*Daphnia*), algae (green algae), and microbes (luminescent bacteria). To confirm that biodegradable materials are not automatically compostable, Gutiérrez et al. [76] analyzed the ecotoxicity of starch/montmorillonite films by examining the growth of the primary root of lettuce (*Lactuca sativa*) seedlings exposed to three different concentrations. They observed that the produced films, besides being marked as biodegradable, revealed to be non-compostable material at high concentrations (100 μg/mL) as measured by their effect on lettuce seedlings.

Salehpour et al. (2018) [17] investigated the effect that the incorporation of cellulose nanofibers (CNF) had on the biodegradation of poly(vinyl alcohol). They conjugate the ASTM D5338 to measure the amount of mineralized carbon, with more extensive analyses of the decomposition process resorting to mass loss, visual, morphology and chemical analysis, and thermal characterization. Moreover, they monitored the impact of the biodegradation in the compost, performing physicochemical and ecotoxicological evaluation by growing cress and spinach. At the end of the assay, it was observed that the PVA/nanocellulose had lower biodegradation rates than the neat PVA. This can be attributed to the difficulty that water molecules suffer to enter the polymer matrix, consequently reducing the microbial growth and activity and to the good interfacial bonding between CNF and PVA that makes it very difficult to break the strong bonds of the film. Another remarkable observation during the thermal analysis was the increase in the biopolymer crystallinity over the composting time. Such can be explained by the evidence that microbes easily degrade the random amorphous component of the polymer, rather than the crystalline phases. Positively, the ecotoxicological assay concluded that no negative chemical effects on the germination and growing of the studied plants were verified during the biodegradation process. 

In the following work, Kalita et al. [63] aimed to study the aerobic biodegradation behavior of modified PLA-based biocomposites under composting conditions by adding to ASTM D5338 other analytical techniques such as molecular weight, differential scanning calorimetry (DSC), contact angle analysis, and microbial colony count. According to the authors, these techniques allow to know how the composting temperature and the change in molecular activity affects the test material wettability. Globally, a decreased trend was observed in contact angles values, suggesting that sample surfaces were becoming more hydrophilic. Meanwhile, as seen in the Salehpour et al. (2018) [17] study, crystallinity percentage increases in biodegraded samples due to loss of amorphous phase at a faster rate, which interestingly counters the previous results because generally contact angles for polymeric materials demonstrate an increasing tendency when the crystallinity percentage is higher. This investigation also offers information about the microorganism counts in compost for each blend. Nutrient colonies agar and plate count agar were the chosen plates to determine colony growth. It was discovered that colony formation was much higher in PLA/Chitosan than in PLA/Nanocellulose and PLA/Gum Arabic. The analyzed plates, using optical microscopy, also demonstrate that gram-negative bacteria were predominant.

It is crucial to recognize the diversity of microorganisms present in compost ecosystems to know which taxonomic class produces the most adequate degradation enzymes to each biopolymer, making the composting more effective. This was the aim that supported the research paper “Tailoring the microbial community for improving the biodegradation of chitosan films in composting environment” by Altun et al. [61]. The work was divided into two parts. In the first place, the authors investigated the biodegradation of chitosan films in controlled composting reactors and analyzed microbial diversity via PCR-denaturing gradient gel electrophoresis (DGGE). Regarding this, it was observed that the dominant taxonomic groups at the phylum level were Ascomycota and Proteobacteria. In the second part, the amount of carbon dioxide emitted by two specific groups of microorganisms added to the inoculum was assessed. The ensemble is composed of Bacillus (*Bacillus circulans*, *Bacillus licheniformis*), Actinobacteria (*Streptomyces roseolus*, *Streptomyces zaomyceticus*), and fungi (*Penicillium islandicum*, *Penicillium chrysogenum*) and were capable of producing chitosanase that increased the chitosan degradation rate. In conclusion, the design and optimization of the microbial community used are important to enhance biodegradation efficiency. In a similar research, Castro-Aguirre et al. [77] isolated and identified the PLA-degrading microbial strains present in compost (*Geobacillus thermoleovorans*) and introduced such strains with bioaugmentation purposes. This is a promising technique used to accelerate the biodegradation of compostable plastics. 

The lack of result reproducibility due to the excessive variability of sources and the different factors that affect biodegradability make it very arduous to evaluate it correctly, whatever the type of environment. However, studies show that performing a complete biodegradation analysis, with controlled and constant monitoring, helps to reduce the error margin. The amount of CO_2_ produced during the process is probably the best indicator of the material’s biodegradability, but this is not enough to evaluate its composting. It is essential to combine this test with others to determine the mechanisms of biodegradation as well as to evaluate its impacts on the environment. Currently, following the guidelines proposed in the ASTM D6400 and ISO 17088, it is already possible to have a good idea of how biopolymers behave under controlled composting situations, but these standards still fail to simulate real conditions. 

### 2.3. Biodegradation Assays in Aquatic Systems 

Plastic litter is the main responsible for the catastrophic scenario of pollution observed nowadays in aquatic systems. This pollutant naturally embarks on its journey in effluents from the wastewater treatment process, reaching inland freshwaters such as rivers and lakes, and then keeps on treading the path to the oceans where it settles and continues disintegrating into micro- and nano-plastics (less than 5 mm). The formed debris may affect the growth, development, behavior, reproduction, and mortality of marine and freshwater fauna. Moreover, the microorganisms existent in the varied waters are suitable hosts for covering the microplastics surface and when ingested by the fauna, they can turn into a public health problem if that food chain reaches humans [80,81]. Few studies evince concern in knowing the mechanisms and conditions of degradation of bioplastics in aquatic systems, most probably because there is still a preconceived idea that they are easily degraded in any context [81,82]. In this circumstance, the scientific community, with research focused on the subject of bio-based plastic, must have as a social responsibility not to transmit the inaccurate idea that the future use of these is seen as an ultimate solution to the problem of marine pollution without causing any harmful effect in the environment. 

The aquatic ecosystem englobes a huge variability of habitats with markedly distinct environmental conditions. Each one influences the biopolymer biodegradability in a particular way, therefore, predicting this feature in aquatic systems is an even more challenging task when compared with terrestrial locations [83]. The water surface, for example, holds more moderate temperatures, receiving UV light from the sun and oxygen from the air, factors that speed up the abiotic degradation. In opposition, the lack of these in deep waters reduces photo- and thermo-oxidative degradation. The hydrolysis rate is equally affected by the type of water, wherever freshwater contains more microorganism concentration than in seawater rich in salt. Some material properties such as density affect the rate of degradation; if the plastic is floating, the degradation is completely different from that of a plastic concentrated in deep waters [84,85]. Within the marine habitat, it is also necessary to consider the geographical location, for instance, the disintegration of a biopolymer in sediments next to where the waves break is not the same as in the open ocean with calm waters [86,87]. Concerning this, biodegradation should be assessed in three significant marine habitats: (a) the pelagic zone, where plastic is floating neutrally buoyantly in the water; (b) the beach sediment zone, which is periodically covered by water due to waves or tide, defined as the eulittoral; (c) the sublittoral benthic zone, where plastic is sunken to the seafloor. 

Currently, the first conclusive studies on the biopolymers biodegradation in aquatic systems are appearing, largely due to the thematic novelty and long duration of the experiment. There is an interconnection between marine and freshwater ecosystems since water derived from waste treatment systems, rivers, and lakes conducts much pollution to the oceans; consequently, the rate of biodegradation of a polymer could be dependent on two different aquatic media [81]. Although, the published results are mainly focused on coastal marine habitats where the residence of plastics is more consistent. It can also be perceived that those studies rarely follow an existing evaluation standard because it is difficult to adapt them to real aquatic conditions. At the present moment, there are no European or international standards focused on studying biodegradation in freshwater systems, and to our knowledge, there is no development for creating one related to inland water bodies. However, regarding marine habitats, some ISO and ASTM standards are available. 

The International Organization for Standardization indicates two norms for the assessment of aerobic plastic biodegradability, which are ISO 18830:2016 “plastics—determination of aerobic biodegradation of non-floating plastic materials in a seawater/sandy sediment interface—method by measuring the oxygen demand in closed respirometer” [88] and ISO 19679:2020 “plastics—determination of aerobic biodegradation of non-floating plastic materials in a seawater/sediment interface—method by analysis of evolved carbon dioxide” [89]. As the name implies, the two methodologies are very similar, centering on experimental laboratory simulations under controlled conditions using seawater and sediments found in different sublittoral benthic zones. The difference between them resides in the biodegradation evaluation, where one assay measures the oxygen demand and the other keeps up the CO_2_ evolution. Furthermore, a standard that determinates ultimate biodegradation in anaerobic conditions can be found under the denomination of ISO 14853:2016 “plastics—determination of the ultimate anaerobic biodegradation of plastic materials in an aqueous system—method by measurement of biogas production” [90]. Two more ISO standards were developed with the intention of simulating a water column scenario, ISO 23977-1:2020 “plastics—determination of the aerobic biodegradation of plastic materials exposed to seawater—Part 1: method by analysis of evolved carbon dioxide” [91] and ISO 23977-2:2020 “plastics—determination of the aerobic biodegradation of plastic materials exposed to seawater—Part 2: method by measuring the oxygen demand in closed respirometer” [92]. The five exposed ISOs are intended for laboratory testing, but the urgent necessity to start methodic testing plastic biodegradation in situ conditions boosted the publication of more standards. To test it in the sea surface ISO 15314:2018 was created, “plastics—methods for marine exposure” [93] and for seafloor and beach scenario ISO 22766:2020 exists, “plastics—determination of the degree of disintegration of plastic materials in marine habitats under real field conditions” [94]. ASTM also proposes three standards about this subject, ASTM D6691-17 “standard test method for determining aerobic biodegradation of plastic materials in the marine environment by a defined microbial consortium or natural sea water inoculum” [95], ASTM D7473/D7473M-21 “standard test method for weight attrition of non-floating plastic materials by open system aquarium incubations” [96], and ASTM D7991-15 “standard test method for determining aerobic biodegradation of plastics buried in sandy marine sediment under controlled laboratory conditions” [97]. The ASTM D6691-17 scope resides in the employment of the measurements of CO_2_ evolution under controlled conditions to determine the degree and rate of aerobic biodegradation of plastic materials to a pre-grown population of at least ten aerobic marine microorganisms living in natural seawater. Complementary to this test, ASTM D7473/D7473M-21 has the objective to predict real-world experiences based on the dimension and tax of biodegradation data for the same test material, bringing into play visual proof of biodegradation and determination of the weight loss as a function of time during the exposure to seawater in a flow-through system. Finally, ASTM D7991-15 [97] simulates the environmental conditions found in the tidal zone, exposing the plastic to collected sediment and seawater. A resume about this extensive information can be revised in the following Table 6. 

The aforementioned standards demonstrate limitations that call into question their reliability when it is intended to be used as a basis for in situ experiments. These limitations were reflected in the excellent review “Biodegradability standards for carrier bags and plastic films in aquatic environments: a critical review” by Harrison et al. (2018) [81], of which we recommend a careful reading. Concisely, these limitations can be cataloged in five different origins: (a) approaches to inoculum preparation and test conditions; (b) absence of specific guidelines for employing different test materials; (c) insufficient statistical replication; (d) a lack of adequate procedures for unmanaged aquatic environments; (f) shortcoming toxicity testing and the impacts of plastic litter on aquatic ecosystems. Furthermore, the arduous climatic conditions often experienced in marine environments, such as storms, strong currents, and waves can compromise the experiment. This experience may also encounter other adversities such as coming into conflict with fishing activities or even hitting recreational boats, which in the last case can lead to sample sabotage and theft. Nevertheless, this type of experimental setup requires exorbitant costs matched only with private financing or grants directed to large-scale projects. This set of factors drives many research groups away from in situ testing and deciding to focus on more simplified laboratory results. This does not mean that it is not possible to make an interesting characterization about the biodegradability of a certain biopolymer in freshwater or seawater, it just becomes more difficult to develop more complex models. This in itself is a significant contribution, orienting future research and proving that more targeted studies are mandatory to directly expose the influence of different biodegradation factors [87,98].

The biodegradation assessment tests that complement the standards, already presented for soil and compost, can equally be applied to aquatic systems. In addition, water absorption, a method to test hydrolytic degradation, is another laboratory test widely used in parallel and which can be useful to predict degradation in systems involving water. The soaked water impregnates the polymer matrix and changes the water gradient in the space between the surface and the inner part of the material [30]. This parameter is crucial to study biopolymers reinforced with fillers, since the reinforcement introduces structural changes in the biopolymer that can affect hydrophilicity. Additionally, the more water the biopolymer is able to absorb, the more likely it is that colonies of microorganisms form. This test also has defined guidelines that can be followed through the ASTM D570-98(2018) “standard test method for water absorption of plastics” [4], ASTM D5229/D5229M-20 “standard test method for moisture absorption properties and equilibrium conditioning of polymer matrix composite materials” [99], or ISO 62:2008 “plastics—determination of water absorption” [100]. Complementarily, solubility analysis can be coupled into the study. The test procedure for the water absorption test is simple, the samples are dried in an oven for an assigned time and temperature and then set in a desiccator to cool. Forthwith, upon cooling the specimens are weighed. The material subsequently emerges in the water at controlled conditions, often 23 °C for 24 h or until equilibrium. Specimens are removed, dried, and weighed. The water intake percentage is the difference in the weight of the specimen before and after the tests. However, 24 h is too short to properly help in the development of a complex biopolymer degradation profile, thus a new experimental design should be created where the standard is adapted to longer times. For instance, Hassan et al. [101] and Jiménez-Rosado et al. [102] observed the hydrolysis degradation during hours, while Kasmuri et al. [103] and Kumar Thiagamani et al. [104] decided to measure it in a question of days. Furthermore, the experiments could use various types of waters, not being limited to distilled water. 

Following the predicate that for the aquatic systems, trusty field test methods and standards for assessing and authenticating biodegradation are lacking; it is crucial to expose two very recent and interesting works. Briassoulis et al. [86] evaluated the standard ISO 19679:2020 [89] and proposed modifications and adjustments to improve the validity and confidence of the methodology in some aspects. Foremost, the authors included agitation in the water surface by a floating magnetic stirrer to simulate wave motion. This modification has the intention of transporting more oxygen from the water surface to the sediments and with that opens the door to fast biodegradation. Generally speaking, they proposed the method should have bigger bioreactors, larger test material quantities mixed with the essential nutrients, and required continuous surface stirring together with a higher threshold. To validate all these alterations, they also recreated field experiments marking the importance of the proposed test to the natural sublittoral conditions. Additionally, Lott et al. [87] presented novel field tests to assess the performance of biodegradable plastics under natural marine conditions. To validate this methodology, it was successfully applied in three coastal habitats (eulittoral, benthic, and pelagic) and in two climate zones (Mediterranean Sea and tropical Southeast Asia). Likewise, they developed a stand-alone mesocosm (or tank) system independent of the direct access to seawater. Mesocosm tests occupy a leading role as a methodological link between field and lab tests because these impersonate a medium that better approximates the real environment than in small-scale laboratory tests. This work is relevant because the outcome has supported the development of the new ISO 22766:2020. 

Two other fresh and remarkable works deserve appreciation in this review. Bagheri et al. [82] designed a one-year comparative degradation study of six different polymers (five taken from the so-called biodegradable polyesters, including poly(lactic-co-glycolic acid) (PLGA), PCL, PLA, PHB, Ecoflex, and one well-known non-degradable polymer poly(ethylene terephthalate) (PET). The polymers were immersed in artificial seawater and freshwater under controlled conditions in a thermostatic chamber at 25 °C and under fluorescence light (16 h light and 8 h dark). Analyzing this study it was possible to conclude that under similar conditions only PLGA presented 100% bulk degradation in both mediums, while PHB, for example, simply showed 8% of degradation after 365 days. The amorphous nature of the polymer could be a possible explanation of the faster hydrolysis and complete degradation of PLGA, making diffusion of water easy all throughout the bulk. Relative to the comparison of the different types of aquatic environments, a similar degradation it was observed but with little tendency to find fast degradation results majorly in freshwater. In the second study, a Japanese team investigated the growth of the bacterial consortium on the bioplastic surface in freshwaters [105]. The authors also isolated and identified the bacteria responsible for that degradation. Briefly, freshwater from 5 different Japanese locations was used to test the biodegradability of 6 distinct bioplastics. The bioplastics were soaked in freshwater inside vial bottles and were incubated at 30 °C with a 150 rpm slow shaking. The formation of significant growth of microorganisms in the bioplastic surface was observed after two weeks. The authors concluded that *Acidovorax* and *Undibacterium* were the predominant genera in most of the samples. 

As a conclusion of this chapter, it is worth recalling that very little is known about the potential of each biopolymer in different aquatic environments, but it is known that degradation is affected by a large number of variables that need to be explored in order to be able to implement novel reliable and more robust research methodologies. 

## 3. Final Remarks

Our society is currently experiencing a period of extreme change due to the urgent focus on combating climate change. Consumers are constantly confronted with new products labeled as natural, biodegradable, or compostable. However, how is this classification achieved? It is essential to create social strategies to educate consumers and companies on how to manage and classify bio-based products in order to minimize the dumping of these residues in inappropriate places where the biodegradation processes would take a longer time or not occur at all and to defy an increasingly established problem in our society, so-called “green-washing”. Thus, the ideas presented in this review summarized the current knowledge about the methodologies that have been adopted to access the biopolymer degradation in different environments (soil, compost, and aquatic systems). Ultimately, the work highlights the uncertainties, difficulties, and existing gaps that still constrain the accurate assessment of the biodegradability of a bio-based plastic entering the market. The information gathered here is valuable to help industrial companies categorize the existing limits to bioplastic degradation and identify potential opportunities to boost the sustainable progress of the food packaging industry towards the production of cleaner and environmentally friendlier packaging, meeting consumers’ and market’s expectations for the future of this important sector of industrial production.

Currently, the global bioplastics sector has presented fast growth because of the continuously emerging range of bio-based and biodegradable polymers production and rising interest in investing in this sector. In a recent report, it was foreseen that its production will expand from 2.11 million tons in 2018 to 2.62 million tons in 2023, with Europe leading the rank of research and development of bioplastics, while Asia stands as a major hub for bioplastic production and consumption [106]. Bio-based industry consortiums in partnership with the European Union (EU) are investing about EUR 3.7 billion on large-scale flagship projects to encourage new technologies in this field. 

Due to the differences in the properties of biodegradable and non-biodegradable polymers, a lot of research is still needed to develop biodegradable polymers or polymer blends/composites that have the necessary properties to replace most of the current non-biodegradable ones. Moreover, when moving toward the goal of widely spreading the production and use of bio-based biodegradable polymers, another constraint arises as a political challenge may occur to educate people to properly dispose of these biodegradable plastics in such a way that they can be transferred to the correct dedicated composting sites for effective biodegradation [107].

When compared with traditional plastics based on non-renewable sources, it is undeniable that the environment benefits from the shorter degradation rates presented by the most diverse bio-based polymers. However, the biodegradation rate is dependent not only on the biopolymer chemical structure but also on the surrounding environmental conditions. Moreover, if other components are incorporated in the bioplastic, such as reinforcement fillers or active agents, it is probable that the biodegradation profile will change. 

Currently, there are no official guidelines to characterize the biodegradation profile of a bio-based polymer; consequently, it is difficult to guarantee that products made from them are labeled correctly. The research teams and certifying companies are compelled to base their results on individual standards used to evaluate conventional plastics. In addition, in many works presented in this review, the results were acquired through the application of ISO and ASTM standards and complemented with other data supported by extra laboratory methodologies. The discrepancy of approaches applied in each work is reflected on the different biodegradability rates identified for the same biopolymer. Therefore, it is urgent to create a policy framework that establishes specifications and validation criteria that fit as a foundation for new biodegradability standards adapted for each specific biopolymer and that can be applied in different environments. Indeed, several procedures can be followed to evaluate the biodegradability of bio-based polymers, two of which are: (a) control of the oxygen consumption or the carbon dioxide production; (b) via determination of mass loss (to evaluate disintegration of the plastic). Yet, it is essential to study the degradation of the biopolymer at various levels. Analyzing the different compounds produced during the decomposition process chemically, and/or analyzing mass loss, and the visual, morphological and thermal characterization, could provide hints on biodegradation mechanisms. Therefore, it is suggested that future standardized protocols to measure bio-base polymer degradation should also be rooted in complementing the standardization with the study of other parameters, namely through chemical analysis of the compounds liberated. 

A bioplastic solely represents a sustainable solution if it is fully biodegradable and if any adverse ecotoxicological effects arise from its degradation. To ensure this, it is fundamental characterization tests are executed not only at the laboratory scale but also in situ through ecotoxicological assessments. Additionally, the microbiota identification present in the ecosystems is also a key aspect to take into consideration in order to determine which taxonomic class produces the most adequate degradation enzymes to each biopolymer. This complementarity requires significant financial effort and is extremely difficult to implement for the majority of the research teams.

It is therefore clear that much remains to be done, and legislators have a key role in establishing standards that help harmonize the biodegradability assays applicable to a biopolymer in order to secure the safety of these new products when distributed in the market.

## Figures and Tables

**Table 5:** Summary of ASTM and ISO standards for plastic degradation in compost.

Standard	Name	Scope
ASTM D5338:15	Standard test method for determining aerobic biodegradation of plastic materials under controlled composting conditions, incorporating thermophilic temperatures	Determination of the ultimate aerobic biodegradability
ISO 14855-1:2012	Determination of the ultimate aerobic biodegradability of plastic materials under controlled composting conditions—method by analysis of evolved carbon dioxide—Part 1: general method	
ISO 14855-2:2018	determination of the ultimate aerobic biodegradability of plastic materials under controlled composting conditions—method by analysis of evolved carbon dioxide—Part 2: gravimetric measurement of carbon dioxide evolved in a laboratory-scale test	
ISO 16929:2021	Determination of the degree of disintegration of plastic materials under defined composting conditions in a pilot-scale test	Determination of the disintegration degree of plastic materials in composting
ISO 20200:2015	Plastics—determination of the degree of disintegration of plastic materials under simulated composting conditions in a laboratory-scale test	
ASTM D6400-21	Standard specification for labeling of plastics designed to be aerobically composted in municipal or industrial facilities	Establishes the requirements for identifying plastics and products made from plastics that compost satisfactorily in industrial and municipal aerobic composting facilities. Includes biodegradation, disintegration, and environmental safety testing requirements and criteria
ISO 17088:2021	Plastics—organic recycling—specifications for compostable plastics	

**Table 6:** Summary of ASTM and ISO standards for plastic degradation in aquatic systems.

Standard	Name	Scope
ISO 18830:2016	Plastics—determination of aerobic biodegradation of non-floating plastic materials in a seawater/sandy sediment interface—method by measuring the oxygen demand in closed respirometer	Determination of the degree and rate of aerobic biodegradation of plastic materials when settled on marine sandy sediment at the interface between seawater and the seafloor by measuring the oxygen demand in a closed respirometer.
ISO 19679:2016	Plastics—determination of aerobic biodegradation of non-floating plastic materials in a seawater/sediment interface—method by analysis of evolved carbon dioxide	Determination of the degree and rate of aerobic biodegradation of plastic materials when settled on marine sandy sediment at the interface between seawater and the seafloor by measuring the evolved carbon dioxide.
ISO 14853:2016	Plastics—determination of the ultimate anaerobic biodegradation of plastic materials in an aqueous system—method by measurement of biogas production	Determination of the ultimate anaerobic biodegradability of plastics by anaerobic microorganisms by exposing the test material to sludge for a period of up to 90 days, which is longer than the normal sludge retention time (25 to 30 days) in anaerobic digesters.
ISO 23977-1:2020	Plastics—determination of the aerobic biodegradation of plastic materials exposed to seawater—Part 1: method by analysis of evolved carbon dioxide	Determination of the degree and rate of the aerobic biodegradation level of plastic materials. Biodegradation is determined by measuring the CO_2_ evolved from plastic materials when exposed to seawater sampled from coastal areas under laboratory conditions.
ISO 23977-2:2020	Plastics—determination of the aerobic biodegradation of plastic materials exposed to seawater—Part 2: method by measuring the oxygen demand in closed respirometer	Determination of the degree and rate of the aerobic biodegradation level of plastic materials. Biodegradation of plastic materials is determined by measuring the oxygen demand in a closed respirometer when exposed to seawater sampled from coastal areas under laboratory conditions.
ISO 15314:2018	Plastics—methods for marine exposure	Description of three methods for the exposure of plastics in a marine environment. Method A covers exposures where specimens float on the surface, method B covers exposures where specimens are partially immersed method C covers exposures where specimens are completely immersed.
ISO 22766:2020	Plastics—determination of the degree of disintegration of plastic materials in marine habitats under real field conditions	Determination of the degree of disintegration of plastic materials exposed to marine habitats under real field conditions. The marine areas under investigation are the sandy sublittoral and the sandy eulittoral zone where plastic materials can either be placed intentionally.
ISO 62:2008	Plastics—determination of water absorption	Determination of the moisture absorption properties in the “through-the-thickness” direction of flat or curved-form solid plastics. Determination of the amount of water absorbed by plastic specimens of defined dimensions when immersed in water or when subjected to humid air under controlled conditions.
ASTM D6691-17	Standard test method for determining aerobic biodegradation of plastic materials in the marine environment by a defined microbial consortium or natural sea water inoculum	Determination of the degree and rate of aerobic biodegradation of plastic materials (including formulation additives) exposed to pre-grown population of at least ten aerobic marine microorganisms of known genera or the indigenous population existing in natural seawater.
ASTM D7473/D7473M-21	Standard test method for weight attrition of non-floating plastic materials by open system aquarium incubations	Determination of the weight loss as a function of time of non-floating plastic materials (including formulation additives) when incubated under changing, open marine aquarium conditions, which is representative of aquatic environments near the coasts and near the bottom of a body of water in the absence of sunlight, particularly UV and visible portions of the spectrum.
ASTM D7991-15	Standard test method for determining aerobic biodegradation of plastics buried in sandy marine sediment under controlled laboratory conditions	Determination of the biodegradation level of plastic materials exposed to laboratory conditions that simulate the environment found in the sandy tidal zone. The tidal zone, that is, the part of the coast affected by the tides and movement of the waves, is the borderline between sea and land, frequently a sandy area that is kept constantly damp by the lapping of the waves.
ASTM D570-98(2018)	Standard test method for water absorption of plastics	Determination of the relative rate of absorption of water by plastics when immersed. This test method is intended to apply to the testing of all types of plastics, including cast, hot-molded, and cold-molded resinous products, and both homogeneous and laminated plastics in rod and tube form and in sheets 0.13 mm (0.005 in.) or greater in thickness.
ASTM D5229/D5229M-20	Standard test method for moisture absorption properties and equilibrium conditioning of polymer matrix composite materials	Determination of moisture absorption or desorption properties in the “through-the-thickness” direction for single-phase Fickian solid materials in flat or curved panel form. Procedures for conditioning test coupons prior to use in other test methods are also covered, either to an essentially moisture-free state to equilibrium in a standard laboratory atmosphere environment, or to equilibrium in a non-laboratory environment. Procedures for determining the moisture loss during elevated temperature testing are also included, as well as moisture loss resulting from thermal exposure after removal from the conditioning environment, such as during strain gauge bonding.

## Data Availability

Data is contained within the article.
